# State and Federal Legislators’ Responses on Social Media to the Mental Health and Burnout of Health Care Workers Throughout the COVID-19 Pandemic: Natural Language Processing and Sentiment Analysis

**DOI:** 10.2196/38676

**Published:** 2023-02-24

**Authors:** Matthew P Abrams, Arthur P Pelullo, Zachary F Meisel, Raina M Merchant, Jonathan Purtle, Anish K Agarwal

**Affiliations:** 1 Department of Emergency Medicine Perelman School of Medicine University of Pennsylvania Philadelphia, PA United States; 2 Center for Digital Health University of Pennsylvania Philadelphia, PA United States; 3 Center for Emergency Care Policy and Research, Department of Emergency Medicine Perelman School of Medicine University of Pennsylvania Philadelphia, PA United States; 4 Leonard Davis Institute of Health Care Economics University of Pennsylvania Philadelphia, PA United States; 5 Department of Public Health Policy & Management School of Global Public Health New York University New York, NY United States

**Keywords:** burnout, wellness, mental health, social media, policy, health care workforce, COVID-19, infodemiology, healthcare worker, mental well-being, psychological distress, Twitter, content analysis, thematic analysis, policy maker, healthcare workforce, legislator

## Abstract

**Background:**

Burnout and the mental health burden of the COVID-19 pandemic have disproportionately impacted health care workers. The links between state policies, federal regulations, COVID-19 case counts, strains on health care systems, and the mental health of health care workers continue to evolve. The language used by state and federal legislators in public-facing venues such as social media is important, as it impacts public opinion and behavior, and it also reflects current policy-leader opinions and planned legislation.

**Objective:**

The objective of this study was to examine legislators’ social media content on Twitter and Facebook throughout the COVID-19 pandemic to thematically characterize policy makers’ attitudes and perspectives related to mental health and burnout in the health care workforce.

**Methods:**

Legislators’ social media posts about mental health and burnout in the health care workforce were collected from January 2020 to November 2021 using Quorum, a digital database of policy-related documents. The total number of relevant social media posts per state legislator per calendar month was calculated and compared with COVID-19 case volume. Differences between themes expressed in Democratic and Republican posts were estimated using the Pearson chi-square test. Words within social media posts most associated with each political party were determined. Machine-learning was used to evaluate naturally occurring themes in the burnout- and mental health–related social media posts.

**Results:**

A total of 4165 social media posts (1400 tweets and 2765 Facebook posts) were generated by 2047 unique state and federal legislators and 38 government entities. The majority of posts (n=2319, 55.68%) were generated by Democrats, followed by Republicans (n=1600, 40.34%). Among both parties, the volume of burnout-related posts was greatest during the initial COVID-19 surge. However, there was significant variation in the themes expressed by the 2 major political parties. Themes most correlated with Democratic posts were (1) frontline care and burnout, (2) vaccines, (3) COVID-19 outbreaks, and (4) mental health services. Themes most correlated with Republican social media posts were (1) legislation, (2) call for local action, (3) government support, and (4) health care worker testing and mental health.

**Conclusions:**

State and federal legislators use social media to share opinions and thoughts on key topics, including burnout and mental health strain among health care workers. Variations in the volume of posts indicated that a focus on burnout and the mental health of the health care workforce existed early in the pandemic but has waned. Significant differences emerged in the content posted by the 2 major US political parties, underscoring how each prioritized different aspects of the crisis.

## Introduction

Health care workers have been disproportionately affected by burnout and mental health symptoms, including depression and anxiety [1-3]. The COVID-19 pandemic has exacerbated mental health symptoms, disorders, and burnout across the workforce [4-14]. Health care workers continue to experience rapid shifts in case volume, critical supply shortages (eg, of personal protective equipment), vaccination rates, death rates, and public health measures [12,15-17]. The emotions and mental health symptoms experienced by workers continue to fluctuate dramatically [14,17,18]. Depression, anxiety, and burnout continue to rise at alarming rates across the health care workforce [19] and have public-facing consequences, such as worse patient outcomes and higher costs [20,21].

State and federal policy responses to the pandemic continue to change across the nation [22,23]. These policy changes have been debated in the public forum by health experts, physicians, and politicians [24]. The link between these policies and case count may lead to hospital-based capacity strain and impact the mental health of the workforce. Ultimately, state COVID-19 policies and political trends are shaping national legislation. For example, President Joe Biden recently signed the Dr. Lorna Breen Health Care Provider Protection Act, inspired by Dr Breen’s death by suicide from the strain of providing care during the COVID-19 pandemic. This reflects how national legislators are starting to recognize the urgent need for improved behavioral health among health care providers.

Social media provides state and federal legislators the opportunity to directly communicate health-related information—including mental health information—to the public and to gauge public interest in a topic [25]. A recent systematic review identified that Twitter can be used to promote public health in 6 main ways, including analysis of shared content and public engagement [26], ultimately informing how governments and health care organizations shape appropriate responses to the COVID-19 pandemic [27]. Social media has also been analyzed to provide insights about the mental health of the general public during the COVID-19 pandemic [28].

The content and language used by state legislators in public-facing venues such as social media reflect their opinions and priorities [23,24,29]. Legislators’ social media posts may also signal attention toward legislation and policy engagement in real time, in addition to their priorities [29-31]. Understanding what policy makers and legislators are saying in these forums is also important, as they have influence over public opinion and impact behavior [32,33]. This may be of particular interest during the COVID-19 pandemic, as US legislators connect with their constituents and influence behaviors related to COVID-19 prevention, safety, and exposure [33,34].

As burnout and mental health symptoms increase among health care workers, the support and opinions of legislators displayed on social media are also important in understanding the message being relayed to the public. Legislators interact on social media broadly, to a greater extent than they share legislative votes or cosponsorship [35]. The growing body of social media exposure on platforms such as Twitter and Facebook between legislators and the general public creates a repository of political opinion and indicators of key policy shifts and messaging. Further, prior studies have found differences reflect a growing divide between Republican and Democratic legislators’ priorities regarding COVID-19 policies [34,36] and, overall, more partisanship than cosponsorship among online interactions between legislators [35]. However, no studies, to our knowledge, have examined possible differences in the views legislators have expressed online regarding the mental health and burnout of the health care workforce.

The objective of this study was to examine state and federal legislators’ social media posts on Twitter and Facebook throughout the COVID-19 pandemic to identify and understand themes related to mental health and burnout of the health care workforce and look for indicators of temporal shifts in political priorities regarding mental health. Specifically, we sought to describe variations in content over time, differences in language and sentiment used across parties, and party-specific theme prevalence. This content is important to analyze in order to understand the public discourse, opinions of the legislature, and the overall response from legislators to burnout and the mental health of the health care workforce.

## Methods

### Data Source

We identified state legislators’ Facebook and Twitter posts related to mental health and burnout in the health care workforce from January 2020 to November 2021 using Quorum (Quorum Analytics) [37], a software platform that collects policy-related documents, including social media content, from politicians during their time in office. For context, there are about 7312 state legislators [38] and 600 federal legislators [39] in the United States. Posts from all members of the upper and lower houses, as applicable, of the 50 US state legislature with 1 or more terms from each of the following keyword groups were selected for analysis: [“healthcare worker,” “doctor,” “physician,” “nurse”] AND [“wellness,” “wellbeing,” “burnout,” “resilience,” “compassion,” “fatigue,” “depression,” “suicide,” “mental health,” “anxiety,” “sad,” “depressed,” “stress,” “stressed,” “tired,” “frustrated,” “frustration”]. Of note, the 4 keywords in the first string of search terms were carefully selected by the research team to capture the health care workers perceived to be most discussed by legislators online and were not inclusive of all frontline workers. Retweets and other posts duplicating the content of another user were also included in the analysis, as these posts indicate the significance of the original content and intent to propagate to a larger audience. This study was conducted in partnership with the Research-to-Policy Collaboration, which is affiliated with Pennsylvania State University's Edna Bennett Pierce Prevention Research Center.

### Descriptive Analysis

Summary statistics were used to describe the volume of relevant burnout-related posts on each social media platform and across parties and legislative bodies. The monthly volume of social media posts related to mental health and burnout between January 2020 and November 2021, stratified by social media platform and political party, was compared with monthly COVID-19 case volume during the same time period. Differences between themes expressed in Democratic and Republican posts were estimated using the Pearson chi-square test. Themes expressed by legislators with independent or unknown affiliations were excluded from the analyses and assessments due to small sample size. Likewise, social media posts from government entities (rather than individual legislators) were excluded from the analyses due to small sample size.

### Natural Language Processing

#### Preprocessing

Post text was converted to lowercase, extraneous white space was stripped, and link URLs, email addresses, user mentions, hashtags, and stop words were removed. Remaining terms were lemmatized to group-inflected forms with the same word stem, and the relative frequency of single words and phrases was extracted to build a baseline set of language features (rows indicated posts, and columns indicated word/phrase frequency), from which the top 50 most frequent words posted by Republicans and Democrats were identified. These methods have been used in prior work characterizing legislator discourse on social media [36,40,41].

#### Theme Modeling

We applied latent Dirichlet allocation (LDA), an unsupervised clustering algorithm, to the baseline set of language features to identify 20 data-driven word clusters (ie, topics) and constructed a topic feature set (rows indicated posts, and columns indicated topic prevalence); LDA assumes that posts have a small number of topics (ie, themes) and that topics are composed of groups of frequently co-occurring words and phrases across posts [42,43]. The topic model was trained using the Machine Learning for Language Toolkit 2.0 [44], and the optimal number of themes was selected via analysis of model coherence scores, visual inspection of topic separation with principal component analysis, and manual evaluation of topic interpretability.

Topic features were correlated (Pearson *r*) with political party (coded as a binary variable, where 0 indicated a Democratic post and 1 indicated a Republican post) to further distinguish linguistic differences across political parties in social media posts about mental health and burnout in the health care workforce. Significant correlations with a Benjamini-Hochberg–corrected *P* value of <.001 and their 95% CIs are reported. Authors AKA and MPA independently evaluated each topic for thematic meaning by reviewing the 10 words and 10 social media posts most associated with each topic [45,46].

#### Sentiment

We applied the Valence Aware Dictionary and Sentiment Reasoner (VADER) [47], a lexicon and rule-based sentiment analysis tool that is specifically attuned to sentiments expressed in social media, to the baseline set of language features to identify weekly changes in post sentiment over time across political parties. Post sentiment scores were calculated as the mean sentence sentiment in each post (as suggested in the VADER documentation), and weekly sentiment scores were calculated as the mean post sentiment for all posts in a given week stratified by party. Sentiment data were visualized via weekly sentiment means overlaid with the exponentially weighted mean of weekly sentiment means. This was repeated to identify monthly changes in sentiment.

All statistical analyses were performed using Python (version 3.7.7).

### Ethical Considerations

This study is exempt from ethical review under University of Pennsylvania Institutional Review Board guidelines, as it does not meet the criteria for human-subject research and utilizes publicly available social media posts.

## Results

The search criteria resulted in 4165 health care workforce burnout–related social media posts, including 1400 tweets and 2765 Facebook posts, that met the inclusion criteria (Table 1).

**Figure 1:**
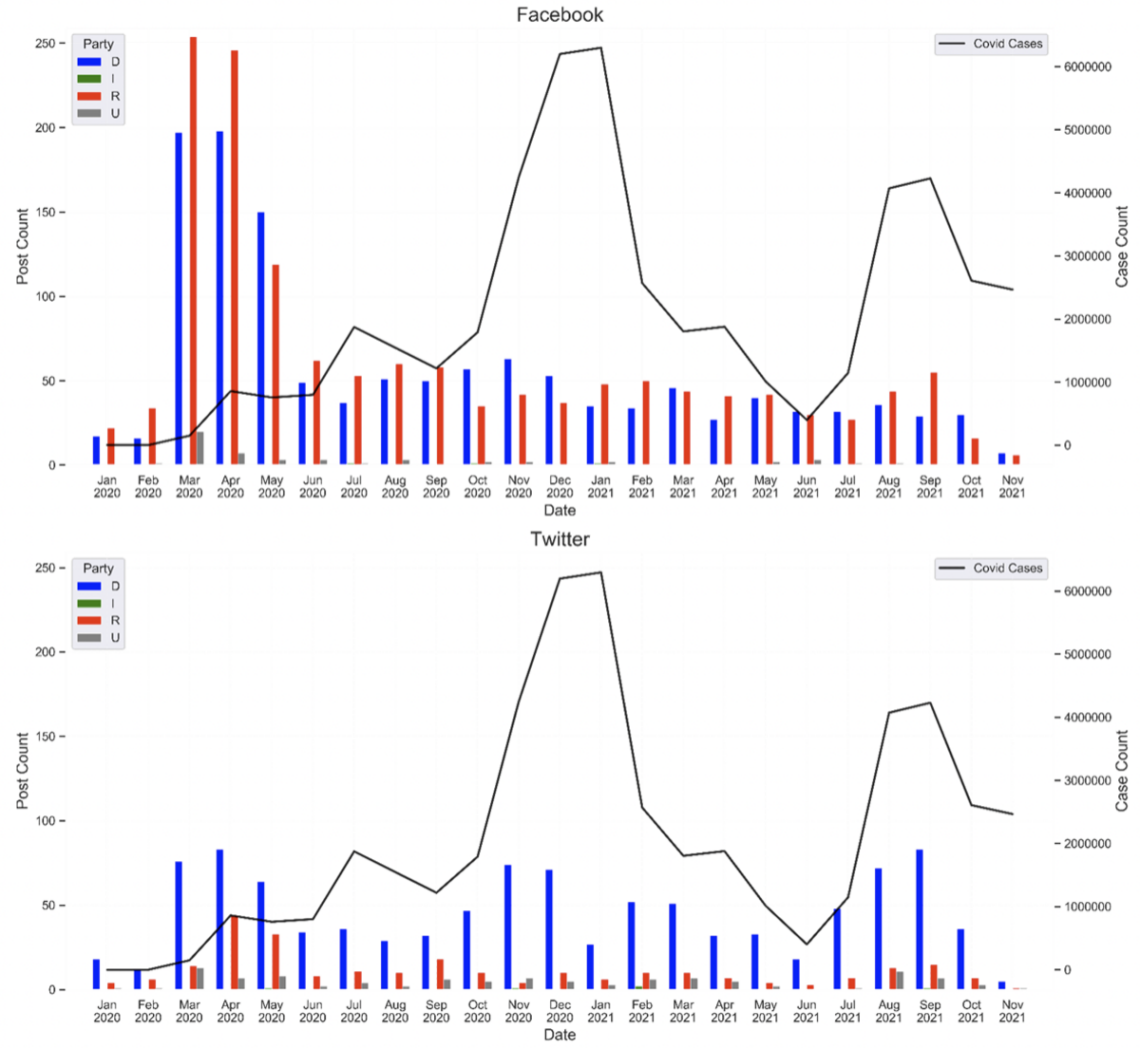
COVID-19 case counts and volume of social media posts by party over time. D: Democratic; I: independent; R: Republican; U: unknown.

These posts were generated by 2047 unique social media accounts, consisting of 2009 state and federal legislator accounts (1257 Facebook accounts and 752 Twitter accounts owned by 1685 unique individuals) and 38 government entity accounts, such as state health departments (n=38 Twitter accounts). The majority of the social media posts (2319/4165, 55.68%) were generated by Democrats. Republicans were responsible for 40.34% (1600/4165) of health care–associated burnout-related social media posts and all other legislators were responsible for 3.58% of posts (166/4165). The most common legislators were representatives (2139/4165, 51.36%) followed by senators (1259/4165, 29.52%). The mean word count was 43.47 (SD 18.87) words for Twitter posts and 422.72 (SD 277.15) words for Facebook posts. Variation in volume of posts generated varied over time, with the majority occurring during the initial surge (Figure 1). This general waning of the volume of burnout-related posts as the pandemic progressed was similar among legislators from both major political parties.

Notable differences were observed between platform use and political party affiliation. Democrats made the majority of Twitter posts (1033/1400, 73.79%) and Republicans made the slight majority of Facebook posts (1425/2765, 51.54%). Additionally, there were notable geographic differences along party lines in the volume of Facebook posts, with Democrats posting more often than Republicans in the Northeast (n=505 vs n=378), and Republicans posting more often from the South (n=727 vs n=418) and Midwest (n=246 vs n=91). However, these regional differences may partially reflect differences in the size and partisan composition of state legislatures across these geographies.

**Figure 2:**
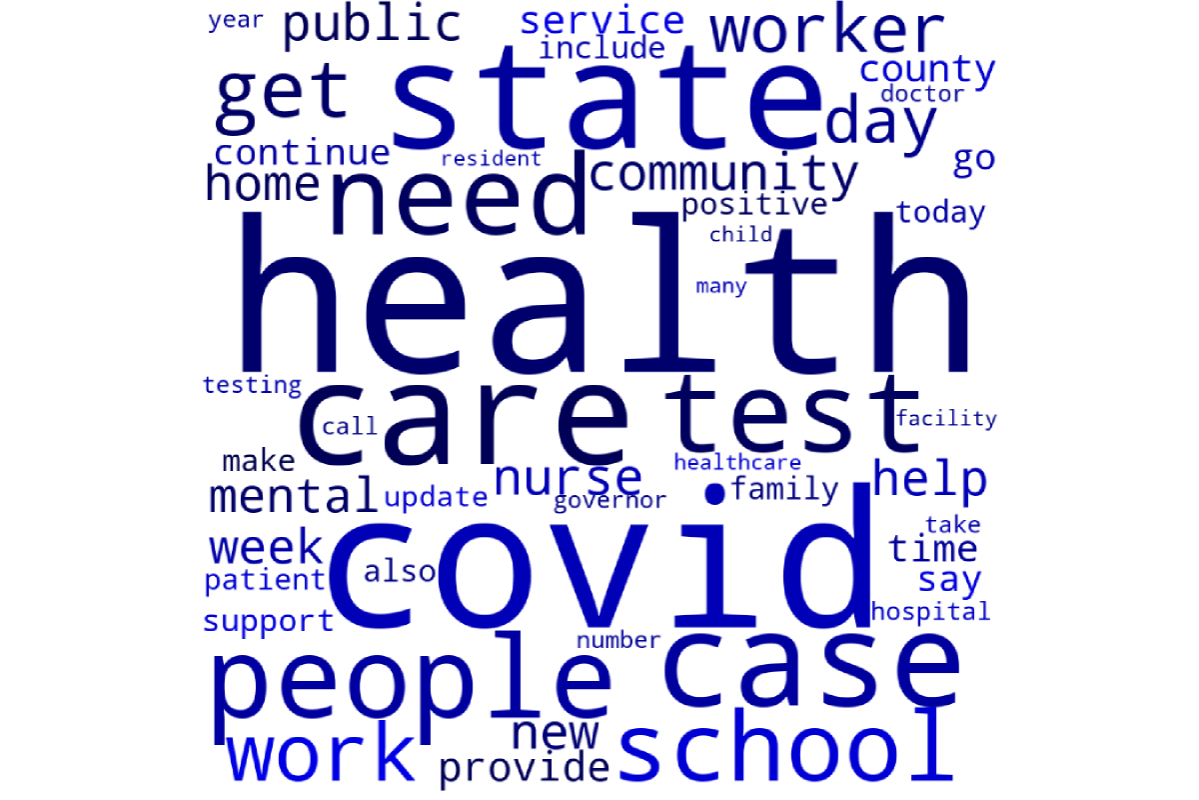
Words most frequently used in Democratic social media posts.

**Figure 3:**
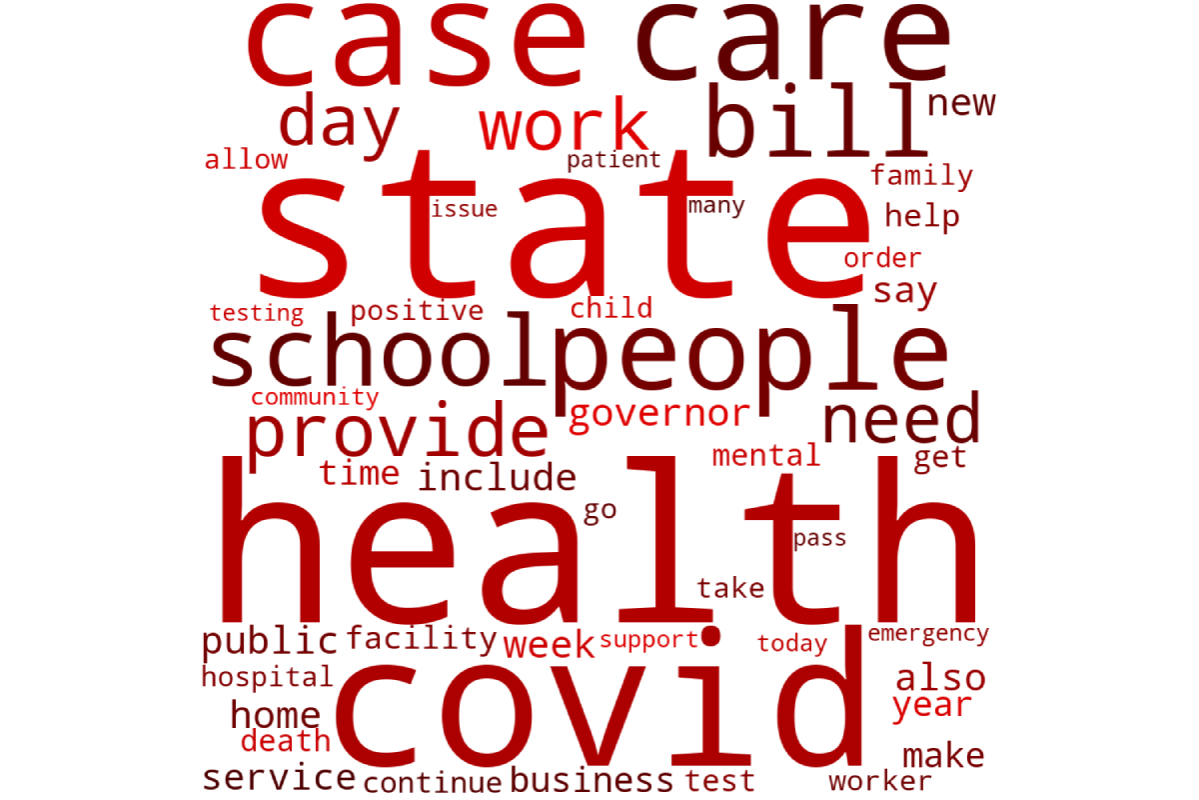
Words most frequently used in Republican social media posts.

Thematic content generated from the natural-language processing and LDA approaches revealed varying content themes between the 2 major political parties (Figures 2 and 3).

The top 4 themes from social media posts most significantly correlated with the Democratic Party were (1) frontline care and burnout, (2) vaccines, (3) COVID outbreaks, and (4) mental health services. The top 4 themes associated with the Republican Party social media posts were (1) legislation, (2) call for local action, (3) government support, and (4) health care worker testing and mental health. Table 2 shows themes, words, and correlation strength with party.

**Figure 4:**
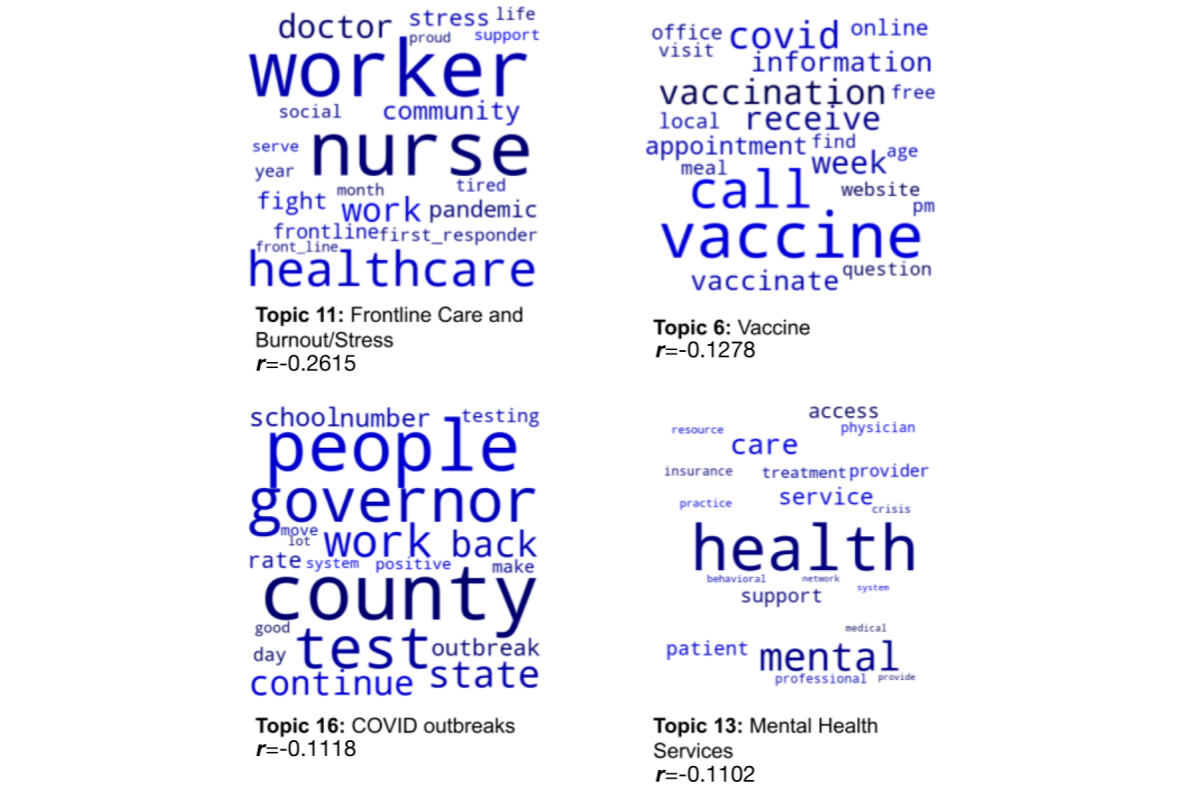
Word clouds representing the top 20 most representative words for each of the 4 themes most correlated with Democratic social media posts.

**Figure 5:**
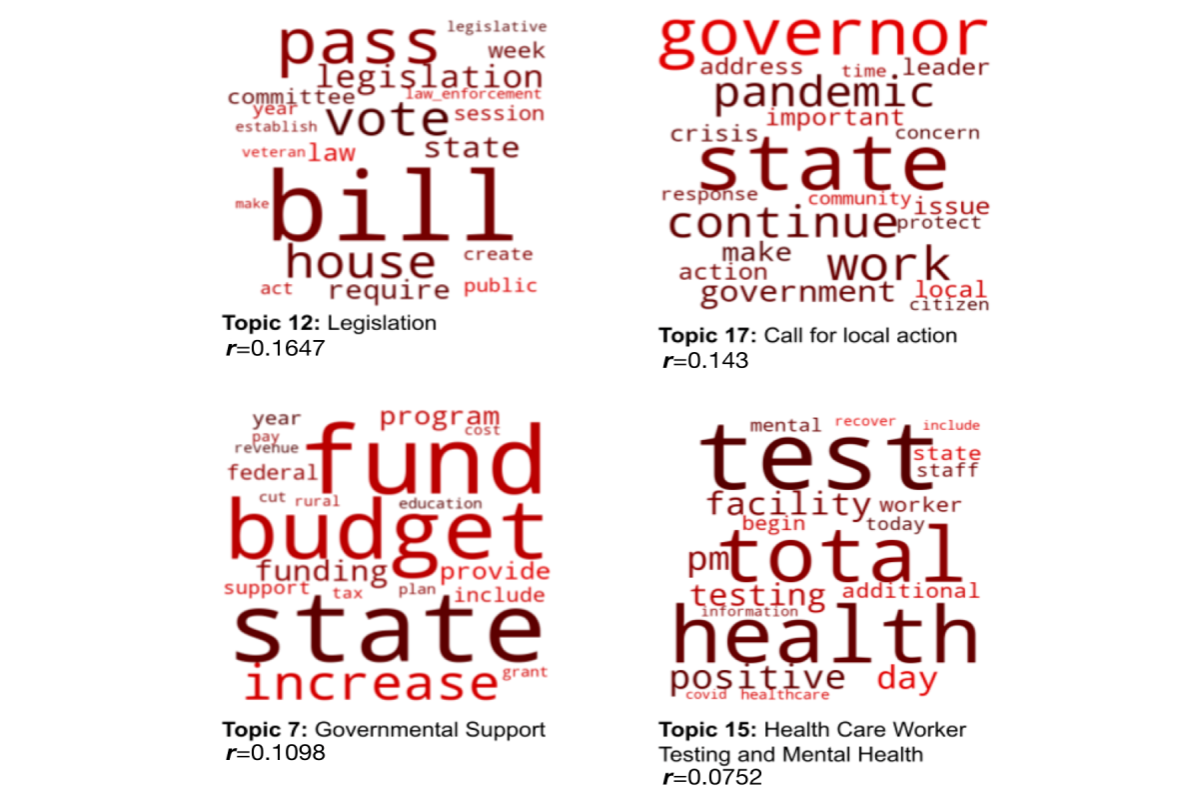
Word clouds representing the top 20 most representative words for each of the 4 themes most correlated with Republican social media posts.

Figures 4 and 5 show word clouds for each of the top 4 themes across party affiliation. Full post content and the list of themes are available in Multimedia Appendix 1, Table S1.

Sentence-level sentiment analyses also revealed differential sentiment patterns by political party throughout the timeline of the study (Multimedia Appendix 1, Figure S1A). The mean monthly post sentiment analysis found that both parties’ posts remained within the slightly positive to positive sentiment range when mean sentiment scores were averaged per month and exponentially weighted. However, the more granular weekly post sentiment analysis by party revealed that during most spikes in COVID-19 case counts, the weekly exponentially weighted mean sentiment scores of Democratic posts more often entered the neutral or negative range compared to Republican posts (Multimedia Appendix 1, Figure S1B).

**Table 1:** Characteristics of social media posts.

Characteristic			Twitter (n=1400), n (%)		Facebook (n=2765), n (%)
**Party**					
	Democratic	1033 (73.79)		1286 (46.51)	
	Republican	255 (18.21)		1425 (51.54)	
	Independent	5 (0.36)		3 (0.11)	
	Unknown	107 (7.64)		51 (1.84)	
**Region^a^**					
	Northeast	445 (31.79)		897 (32.64)	
	South	365 (26.07)		1170 (42.58)	
	Midwest	285 (20.36)		446 (16.23)	
	West	304 (21.71)		235 (8.55)	
**Status**					
	Current	1338 (95.57)		2535 (91.68)	
	Designate	1 (0.07)		3 (0.11)	
	Former	61 (4.36)		227 (8.21)	
**Government title^b^**					
	Representative	623 (22.53)		1516 (54.83)	
	Senator	439 (15.88)		820 (29.66)	
	Assembly	115 (4.16)		158 (5.71)	
	Delegate	54 (1.95)		118 (4.27)	
	Governor	54 (1.95)		73 (2.64)	
	Speaker	9 (0.33)		40 (1.45)	
	Member	0 (0)		14 (0.51)	
	Other	18 (0.65)		26 (0.94)	

^a^Posts from Guam, the Virgin Islands, and the Northern Mariana Islands were not included.

^b^Posts from government entities (n=88 Twitter posts) were not included.

**Table 2:** Themes associated with Democratic or Republican posts.

Theme		Top words	Pearson *r* (95% CI)
**Themes associated with Democratic posts**			
	Frontline care burnout and stress	worker, nurse, healthcare, work, doctor, community, fight, stress, pandemic, frontline, first_responder, social, tired, year, life, month, support, serve, front_line, proud	–0.2615^a^ (–0.31 to –0.21)
	Vaccine	vaccine, call, covid, vaccination, receive, week, information, vaccinate, appointment, online, local, office, meal, website, free, visit, find, pm, question, age	–0.1278^a^ (–0.18 to –0.08)
	COVID outbreaks	county, people, governor, test, work, state, back, continue, school, number, outbreak, rate, testing, day, make, positive, move, system, good, lot	–0.1118^a^ (–0.16 to –0.06)
	Mental health services	health, mental, care, service, access, support, patient, provider, treatment, physician, professional, insurance, crisis, practice, resource, behavioral, medical, provide, system, network	–0.1102^a^ (–0.16 to –0.06)
	COVID testing	covid, testing, health, information, state, include, public, update, test, site, department, community, today, member, resident, contact, resource, day, announce, visit	–0.0582 (–0.11 to –0.01)
	State information	case, covid, county, health, statewide, update, coronavirus, individual, state, home, death, total, patient, provide, stay, information, report, number, continue, resident	–0.0398 (–0.09 to 0.01)
	Schools and education	school, child, student, education, year, district, teacher, high, parent, family, plan, learn, person, work, board, adult, college, staff, opportunity, ensure	–0.0348 (–0.09 to 0.02)
	Masking to slow spread	virus, people, spread, mask, risk, coronavirus, medical, disease, sick, doctor, prevent, stay, condition, symptom, show, time, flu, avoid, slow, wear	–0.0243 (–0.08 to 0.03)
	Frontline/essential service support and volunteers	service, include, provide, medical, support, public, community, food, individual, provider, essential, work, worker, center, company, care, supply, volunteer, health, equipment	–0.0116 (–0.06 to 0.04)
	Family/support systems	worker, nurse, healthcare, work, doctor, community, fight, stress, pandemic, frontline, first_responder, social, tired, year, life, month, support, serve, front_line, proud	–0.0019 (–0.05 to 0.05)
**Themes associated with Republican posts**			
	Legislation	bill, pass, vote, house, legislation, state, require, law, week, committee, session, public, year, create, act, law_enforcement, veteran, legislative, establish, make	0.1647^a^ (0.11 to 0.21)
	Call for local action	state, governor, work, continue, pandemic, government, make, issue, important, address, action, local, crisis, leader, response, concern, protect, community, citizen, time	0.1430^a^ (0.09 to 0.19)
	Governmental support	state, fund, budget, increase, funding, program, provide, federal, include, year, support, tax, grant, cut, plan, education, pay, revenue, cost, rural	0.1098^a^ (0.06 to 0.16)
	Health care worker testing and mental health	test, health, total, positive, pm, facility, day, testing, additional, worker, state, begin, staff, mental, today, healthcare, recover, information, include, covid	0.0752^a^ (0.02 to 0.13)
	Business/economy	business, order, home, public, health, stay, guidance, follow, close, guideline, essential, open, social_distance, employee, issue, reopen, continue, activity, remain, limit	0.0676^a^ (0.02 to 0.12)
	Pandemic time course	time, day, people, make, work, give, place, put, today, good, week, call, happen, start, month, understand, long, point, post, end	0.0675^a^ (0.02 to 0.12)
	Emergency public health measures	emergency, state, provide, program, public, benefit, federal, assistance, business, payment, requirement, covid, extend, pay, department, governor, sign, receive, apply, require	0.0379 (–0.01 to 0.09)
	Debate surrounding public policies	woman, mandate, decision, government, protect, policy, force, doctor, fail, power, lead, lose, sadly, life, abortion, hearing, drug, speak, freedom, science	0.0302 (–0.02 to 0.08)
	Case counts	case, death, positive, covid, active, test, change, report, yesterday, number, week, hospital, total, bed, patient, update, day, increase, confirm, rate	0.0268 (–0.03 to 0.08)
	Long-term care facilities	care, facility, home, family, nursing, health, hospital, resident, nursing home, staff, visit, long-term, member, patient, visitation, vulnerable, person, senior, hour, individual	0.0197 (–0.03 to 0.07)

^a^These values were significant at the *P*<.001 level after applying the Benjamini-Hochberg correction for multiple tests.

## Discussion

### Principal Findings

This study investigated the social-media posts of US legislators throughout the COVID-19 pandemic with a focus on content related to health care–associated burnout and the mental health of the workforce. It has 3 key findings. First, state and federal legislators are actively using social media to discuss the pandemic and burnout. Second, the focus on burnout and the mental health of the health care workforce was primarily seen in the early surge of the pandemic and then dramatically waned. Third, key differences emerged in the social media content posted by the 2 major US political parties. Addressing the overlapping nature of the COVID-19 pandemic and health care–associated burnout is a national priority for health systems, payers, clinicians, and patients [7], yet the 2 parties appear to highlight and prioritize different aspects of the crisis.

State and federal legislators are increasingly using social media as a platform to discuss health care and medicine [31,35,36,40]. Previous literature has investigated the relationship between Democrats’ and Republicans’ social media content within the context of the opioid epidemic, showing that overall partisanship across topics increased from 2016 to 2019 [40]. In the setting of the COVID-19 pandemic, a recent study also showed that Republican legislators who were previously less engaged in discussion of vaccination on social media became significantly more publicly engaged following the arrival of COVID-19 compared to their Democratic counterparts, suggesting a possible convergence of priorities in light of the COVID-19 pandemic [41]. The content posted on Twitter and Facebook is public facing, and given the rise of digital technology and social media, the content posted by legislators in the United States provides a window into political thoughts, agendas, and priorities. The pandemic has certainly worsened the mental health strain and burnout faced by health care providers and is projected to continue despite improvements in case volume [7]. This is among the first studies to discover and investigate the social media content from US legislators specific to burnout and mental health of the workforce. Perhaps less surprising is the rise in these social media posts early in the pandemic, as attention was keenly focused on the workforce. Unfortunately, this data set shows that after the initial wave, there has been less attention over time despite recurrent surges (eg, Delta variants). In line with Kingdon’s multiple streams model [48], this may indicate that the “policy window” for mental health–related legislation regarding the health care workforce was open early in the pandemic. That said, there remains a persistent, yet small, discussion across parties, but ultimately it is low.

The themes and words that state and federal legislators used in these mental health–related social media posts were notably different between the 2 major political parties, including in their emphasis. This is consistent with another recent analysis of tweets from legislators that found differences in health care–related themes according to party lines [36]. In our study, Republican-affiliated legislator posts revealed a greater representation of themes central to public policies and legislation. The themes indicated a focus on local and federal action as seen through 2 of the top 4 most strongly correlated themes, “call for local action” and “governmental support.” This may reflect support for implementing broader policies to help support health care workers. Republican posts also included a focus on COVID-19 testing for the workforce. In contrast, Democratic social media posts more specifically focused on the mental health services and acute strain on the workers themselves. The thematic analysis showed that 2 of the top 4 themes focused on “frontline care and burnout/stress” and “mental health services.” In addition, Democratic posts were varied in their overall content, with other themes emerging related to capacity strain on health systems related to outbreaks and vaccines and vaccinations themselves. These themes appear to be much more granular and focused on the workers themselves and the stress and burnout they face throughout the pandemic surges.

This is among the first studies to use natural language processing of state and federal legislators’ social media content to measure and describe trends in content and posting issues over time with specific attention to health care worker burnout and mental health. State and federal legislators’ word choices on social media carry great influence, and their reach is broad. The posts generated by legislators reflect the immense initial concern and the seeming loss of focus as the public response evolved over the course of the pandemic. Discussing mental health and burnout in public forums is important in health care, where significant stigmas exist and the consequences are grave, as seen by the high relative rate of physician suicide [49-51]. State and federal legislators carry power in their voices, whether they are live or on social media, and their words can lead to important action to help support and sustain the workforce. Recognizing the urgent need for improved behavioral health among health care providers, President Joe Biden recently signed the Dr. Lorna Breen Health Care Provider Protection Act, inspired by Dr Breen’s death by suicide from the strain of providing care during the COVID-19 pandemic. Highlighting the important role of legislators’ social media, the post on the President’s Instagram account (@Potus) about this new act’s aim of “reducing and preventing suicide, burnout, and mental health and substance use conditions among healthcare professionals” received over 330,000 likes and 7200 comments, suggesting social media is an important tool for legislators to interface with constituents about the mental health of the workforce.

### Limitations

This study has several limitations. Quorum does not report state or federal legislators’ years in office, only whether they are a current or former legislator at the time of data download. We therefore were unable to stratify for years in office in our measures of legislators’ number of social media posts related to burnout or mental health. Similarly, Quorum does not report the gender of legislators. It is possible that the content may be different based on the gender of legislators, so future studies should aim to analyze legislators’ posts by gender. We also did not have access to the total number of social media posts for each legislator. We were therefore also unable to stratify for a legislator’s general social media activity in our analysis. Another limitation is that social media posts from both state and federal legislators were aggregated and analyzed together. However, it is possible that variations in the content and sentiment of social media posts may differ based on whether a legislator works at the state or federal level.

Moreover, cross-party comparisons in post volume are impacted by the size and partisan composition of state and federal legislatures, which are often not evenly distributed along party lines; therefore, regional differences in attention to burnout within these geographical regions should be interpreted with caution, since there may be different numbers of Democratic versus Republican legislators in a given region. Another limitation is that changes in the content of social media posts in relation to major changes in pandemic prevention and control, such as lockdowns, the introduction of vaccines, vaccine mandates, and masking, were not considered in the analyses. Given it is possible that the content in posts may vary based on these major events, more granular analyses that look at how social media content was influenced by prevention efforts should be conducted in the future.

Finally, social media language does not necessarily lead to specific votes or policy decisions. Identifying relationships between state and federal legislator social media content and legislator voting patterns was beyond the scope of this project.

### Conclusion

Health care–associated burnout and mental health strain has grown tremendously throughout the pandemic. Public and legislative response and attention is key to ensuring those working in health care are supported and cared for, as burnout impacts clinicians and the care they provide. Social media can provide valuable insight into trends in state and federal legislators’ burnout and mental health–related content. We found an initial surge in the volume of posts that has diminished throughout the pandemic and, perhaps unsurprisingly, a divide in how Democrats and Republicans think about the issues. Democrats increasingly post content related to individuals and stress and Republicans increasingly post content related to legislation. As the pandemic case count diminished, we found an unfortunate similar decrease in attention from legislators to the issue of supporting the mental health of health care workers and combating burnout.

## Appendix

Multimedia Appendix 1 Supplemental Figure 1: Mean Social Media Post Sentiment Scores by Political Party Affiliation and Supplemental Table 1: Representative Social Media Posts Associated with Each Theme.

## Abbreviations

LDA: latent Dirichlet allocation
VADER: Valence Aware Dictionary and Sentiment Reasoner

## Data Availability

The data sets generated during and/or analyzed during the current study are available publicly from the Quorum database, as well as from the corresponding author on reasonable request.
