# Supplementary material for: State and Federal Legislators’ Responses on Social Media to the Mental Health and Burnout of Health Care Workers Throughout the COVID-19 Pandemic: Natural Language Processing and Sentiment Analysis
Source: JMIR Infodemiology. 2023 Feb 24;3:e38676. doi: 10.2196/38676 (PMC10007003; doi:10.2196/38676)
Supplement: Multimedia Appendix 1 [file infodemiology_v3i1e38676_app1.docx]

**Supplemental Figure 1:** Mean Social Media Post Sentiment Scores by Political Party Affiliation

**1A:** Mean Monthly Sentiment Scores of Social Media Posts by Political Party

**
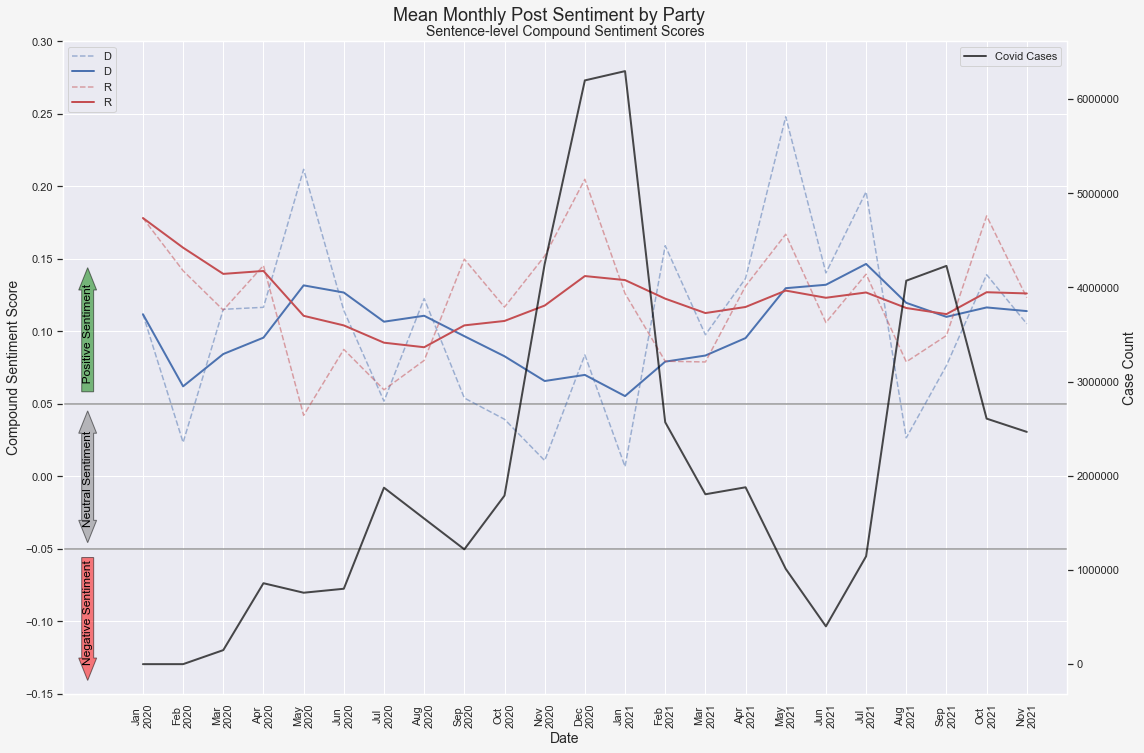
**

**1B:** Mean Weekly Sentiment Scores of Social Media Posts by Political Party

**
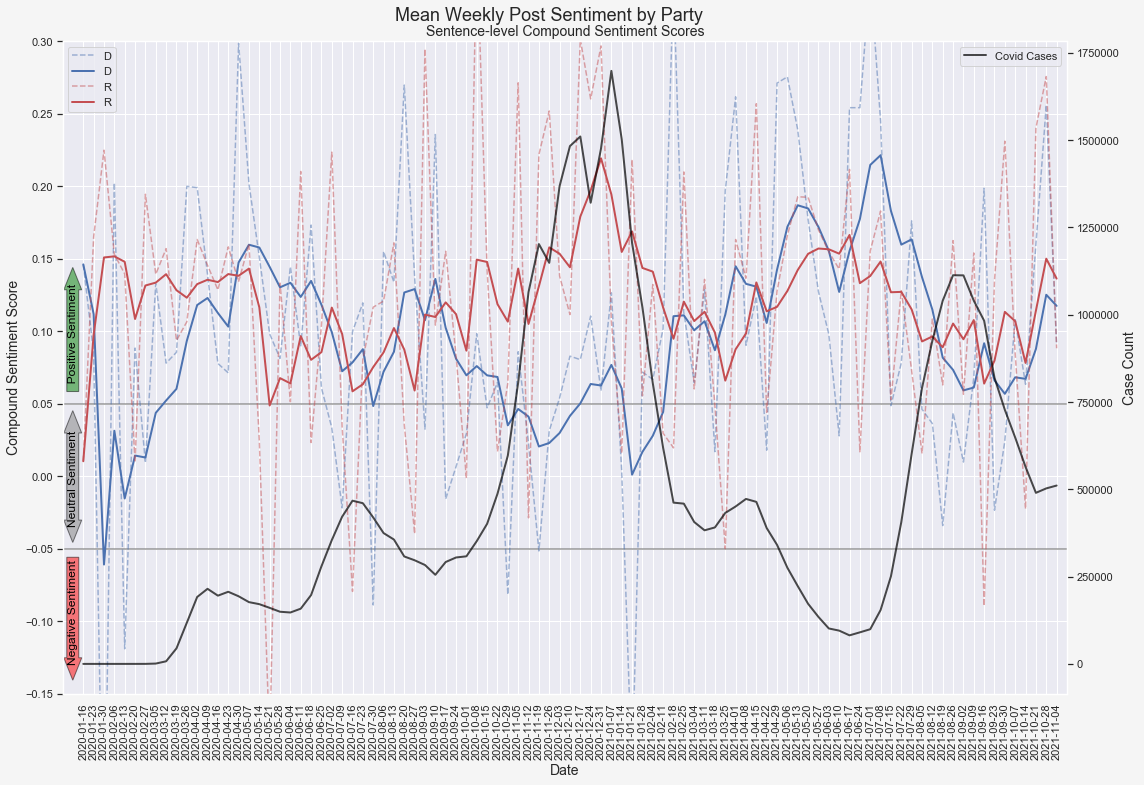
**

**Figure Legend**:

1. The dotted blue/red lines show the monthly/weekly sentiment scores for each post. The scores were calculated by taking the mean of the compound sentiment score of each sentence in the post. Monthly or weekly sentiment scores were then calculated as the mean of post sentiment scores over the time interval.
2. The solid blue/red lines show the exponentially weighted means of sentiment scores
3. The solid black line shows raw Covid-19 case counts
4. The horizontal grey lines and colored arrows show the boundaries for positive, neutral, and negative sentiment scores

**Supplemental Table 1:** Representative Social Media Posts Associated with Each Theme

| **Theme** | **Representative Social Media Posts** |
| --- | --- |
| **Frontline Care and Burnout / Stress** | *As the numbers in NJ continue to tick up I ask everyone to try to have empathy, compassion and understanding for those around them. Have empathy and compassion for our doctors, nurses and health professionals who unfortunately find themselves in Round 2 of sleepless nights, increased patient loads and uncertainty for their own futures. Have empathy and compassion for our police, firefighters and first responders who put their lives on the line in the midst of a pandemic for us. Have empathy and compassion for our most vulnerable in our Long Term Care Facilities who may be spending their last or close to last holidays alone, without the ability to celebrate with their families….Have empathy and compassion for those who are frustrated, depressed or struggling with the realization that we are not yet out of this. Have empathy and compassion for those that may not share the exact same beliefs and philosophies as you may. Be grateful if you've had the ability to not worry about your job, your mortgage, your business, your children. And most importantly understand that for most the past year has been incredibly difficult in so many ways. Stop judging. Understand that your way of coping or dealing may be far different from someone else's reality. We will eventually emerge from this surreal year but now is the time to be smart. Wear your mask. Wash your hands. Use common sense and please stay safe.* |
| **Vaccine** | *VACCINE UPDATE from the Oregon Health Authority: Older adults age 65 and above will become eligible to get a vaccine over the following 3 weeks. Here's information for you to help reduce confusion and frustration as we work together to support Oregon seniors at a time when vaccines remain in critically short supply in the United States and here in Oregon. Older adults will have different ways to get vaccine information Vaccines are scarce. In coming weeks, we know there will be more seniors who want to get vaccinated than there will be vaccines available to them. While we can't give every older adult an appointment as soon as they want one, we can give them different ways to get linked to vaccine information and events. Here are some of the options that will be available to all seniors on February 8: * Getvaccinated.oregon.gov web tool: This tool will allow people to determine if they are eligible for a vaccine and register to get email alerts or text notifications about vaccine events in their area. The Get Vaccinated Oregon tool will be open to all Oregonians and information will be available in 11 languages. o The getvaccinated.oregon.gov URL is not yet live! We will notify you when the URL goes live in coming days. * 211: Older Oregonians can also text ORCOVID to 898211 to get text/SMS updates (English and Spanish only) or email ORCOVID@211info.org. If they can't get their COVID-19 vaccine question answered on the website, by text, or by email, they can call 211 or 1-866-698-6155, which is open from 6 a.m. to 7 p.m. daily, including holidays.…Pharmacy partners are vaccinating residents and staff at senior residences and care facilities. Residents (or family members) can ask their facility operator when their residence is scheduled for a vaccination clinic. Every senior will be able get a vaccine, but most seniors will not be able to get immunized for many weeks There are more than 750,000 people aged 65 or older. While Oregon has already vaccinated more than 100,000 people aged 60 and older, state health officials estimate it take until mid-April to vaccinate more than 7 in 10 Oregon seniors. All seniors will be eligible to get vaccinated by the end of February…* |
| **COVID outbreaks** | *Governor's Update Wednesday, 9.16.20 Governor Justice * 10 additional deaths in WV; bringing the death toll to 290. * Daily positive rate: 5.61%. Cumulative rate: 2.67%. Hospitalized- 156, up from 155. Rt rate has dropped to 1.22- third worst in the nation. * 220 positive new cases in in last 24 hours; 3,235 current active cases * Kanawha and Mon County are turning red today; Orange- Putnam, Fayette, Boone, and Mingo; Gold- Ohio, Calhoun, Pocahontas, Cabell, Lincoln, Logan * Solved the riddle of daycare- committing another $6M to continue the program of funds for essential workers to receive daycare through the end of the year, believes there's a chance that Federal money will come through and backfill but even still the money is there to continue program… Also, to make sure that we are not reducing the drive to test more, the more test the more we identify and can keep the spread down * Looking at the communities with colleges and universities helps to see how to be flexible and more decisively define the spread and rates in the community, determine which students are most high at risk of spread * Goal is to bring more testing to gold, orange, and red counties - will also help to bring numbers down because of the negative vs. the positive results Dr. Lee Smith * Mon County is still a ruralized county with centralized populations, specifically Morgantown, analyzing data since July * 20-29 age group is vast majority of the positive numbers and this is mostly Morgantown, not in the more rural areas * Concern for spread from university to general population, and the data is not showing that being born out- looking to the bordering counties are not seeing similar rises of COVID cases in their population, lots of people employed from these counties and are not seeing spread * WVU has stepped up and recognized their role and providing a secure and monitored location for their students; feel that the county should treat this as a single outbreak because of congregate setting and addressed like they would a nursing home, and think this is a model that can be used in other counties with institutions of higher ed Chancellor Sarah Tucker * Less than 1% of students across twenty colleges have positive test cases, thanks Governor and local health departments for having students tested prior to going back * Safest way to address positive cases in students is to keep the students on campus to address the needs, colleges are following guidelines of ACHA and setting up isolation housing…Working with DHHR and National Guard to create an ongoing plan for surveillance tests of students and staff to continue to monitor safety and health Governor Justice * Reiterates the value of getting tested; also recognizes that this is the best way for the communities to be able to do more things by showing infection rate and this brings the rates down * …* |
| **Mental Health Services** | *….On Thursday, April 9, President. Trump, Vice President Mike Pence, First Lady Melania Trump, and Second Lady Karen Pence spoke by telephone with hundreds of American mental health professionals, leaders, and advocates to discuss the effects COVID-19 is having on the American people. This COVID-19 call was the first to bring these four influential leaders together on one issue. The President recognized that a great number of Americans are enduring hardships - including loneliness from social distancing, despair from being out of work, anxiety from the danger of the virus, and grief from the loss of a loved one. President Trump thanked the doctors, counselors, and many other mental health professionals for providing vital mental health services during this time through tele-health. U.S. Surgeon General Jerome Adams PSA - Mental Health The White House and Administration are committed to providing Americans with vital mental health resources and services especially through expanding telehealth services, continuing the Administration's efforts to combat drug demand and the opioid crisis. Below, please find additional information and resources. Also find a helpful Fact Sheet from the White House Office of National Drug Control Policy (ONDCP) outlining efforts and resources to ensure that prevention, treatment, recovery support services, and safe and effective pain management remain available nationwide. We also want to share examples of State and Local leaders addressing mental health needs in their communities and welcome awareness and engagement around your efforts.…*  *STATE AND LOCAL COVID-19 MENTAL HEALTH INITIATIVES Below are examples of State and Local leaders promoting mental health awareness and resources aid the COVID-19 pandemic. We welcome awareness over your own respective initiatives. Ohio Governor DeWine urged support groups to check in with people with serious mental illness or addiction and highlighted the need to get the suicide hotline sent out to more people. Rhode Island Governor Gina Raimondo offered resources to those struggling with mental health during this time. Tennessee Governor Bill Lee addressed the collateral impact on mental health that COVID-19 takes and highlighted the state's crisis hotline while working with the Tennessee Department of Mental Health and Substance Abuse Services to expand telehealth access. Indiana Governor Eric Holcomb said that mental health crisis calls are increasing and will issue guidance for Hoosiers on the tools available to help them out with mental health issues and will increase staffing at these centers. Minnesota Governor Tim Walz signed an Executive order expanding mental health counseling options for Minnesotans. Specifically, the Executive Order authorized out-0f-state mental health providers to treat Minnesota patients via telehealth services Illinois Governor J.B. Pritzker announced the launch of a new Remote Patient Monitoring Program and mental health support line. Call4Calm, operated by the Illinois Department of Human Services' Mental Health Division, is a free-of-charge emotional support text line for Illinois residents experiencing stress and mental health issues related to COVID-19. The Michigan Department of Health and Human Services launched a warmline to help Michiganders with persistent mental health issues amid the COVID-19 pandemic. The peer-operated warmline is intended to act as a resource for people experiencing depression, anxiety, or other mental illnesses. The Texas Health & Human Services Commission established a hotline and texting service on Mental Health. San Diego, California Mayor Kevin Faulconer and San Diego County Supervisors announced a $25 Million partnership for a Behavioral Health Fund dedicated to assessment, treatment, detoxification, crisis stabilization, residential treatment and supportive housing. San Jose, California Mayor Sam Liccardo hosted a Facebook Live session on mental health during the coronavirus crisis. The Los Angeles County Department of Mental Health (DMH), the largest county mental health department in the United States, has scaled resources and provided coronavirus-specific guidance for individuals during the pandemic…* |
| **COVID testing** | *Good morning, Clay County COVID-19 Hotline: 877-252-9362 This local resource is available Monday-Sunday, 8 AM-5 PM. The latest updates from the Governor's Office: FOR IMMEDIATE RELEASE March 22, 2020 ~768 positive cases in Florida Residents and 62 positive cases in non-Florida residents TALLAHASSEE -- To keep Florida residents and visitors safe, informed and aware about the status of the virus, The Florida Department of Health has launched a COVID-19 dashboard that will be updated twice daily. Today, as of 11 a.m., there are 830 total** Florida cases One person has died who tested positive for COVID-19 in Palm Beach County. New Florida cases include: * 67 additional positive COVID-19 cases (62 Florida residents and 5 non-Florida residents) reported to the Florida Department of Health. * There are currently 768 positive cases in Florida residents and 62 positive cases in non-Florida residents. Florida recently partnered with private laboratories around the state to expand COVID-19 laboratory testing capacity. This partnership will increase the number of tests conducted each day and ensure Floridians receive the critical health information they need in a timely manner. Expansion to private laboratories changes the COVID-19 testing landscape in Florida. Private laboratories are running tests as they receive swab samples from practitioners. Testing and reporting times vary among commercial and DOH laboratories. Demographic information may be updated during investigations. These twice daily reports reflect the state's efforts to accurately and transparently share information.… Florida recently partnered with private laboratories around the state to expand COVID-19 laboratory testing capacity. This partnership will increase the number of tests conducted each day and ensure Floridians receive the critical health information they need in a timely manner. Expansion to private laboratories changes the COVID-19 testing landscape in Florida. Private laboratories are running tests as they receive swab samples from practitioners...* |
| **State Information** | *…Department of Health Provides Update on COVID-19, 885 Positives Bring Statewide Total to 42,050 Harrisburg, PA - The Pennsylvania Department of Health today confirmed as of 12:00 a.m., April 27, that there are 885 additional positive cases of COVID-19, bringing the statewide total to 42,050. All 67 counties in Pennsylvania have cases of COVID-19. Today, the state is reporting 1,597 deaths in Pennsylvania. County-specific information and a statewide map are available here. All people are either in isolation at home or being treated at the hospital. "As we see the number of new COVID-19 cases continuously change across the state that does not mean we can stop practicing social distancing," Sec. of Health Dr. Rachel Levine said. "We must continue to stay home to protect ourselves, our families and our community. If you must go out, please make as few trips as possible and wear a mask to protect not only yourself, but others. We need all Pennsylvanians to continue to heed these efforts to protect our vulnerable Pennsylvanians, our health care workers and frontline responders." There are 161,372 patients who have tested negative to date. Of the patients who have tested positive to date the age breakdown is as follows: Nearly 1% are aged 0-4; Nearly 1% are aged 5-12; 1% are aged 13-18; Nearly 6% are aged 19-24; 38% are aged 25-49; Nearly 28% are aged 50-64; and Nearly 26% are aged 65 or older. Most of the patients hospitalized are aged 65 or older, and most of the deaths have occurred in patients 65 or older. There have been no pediatric deaths to date. More data is available here. In nursing and personal care homes, there are 7,037 resident cases of COVID-19, and 862 cases among employees, for a total of 7,899 at 441 distinct facilities in 40 counties. Out of our total deaths, 990 have occurred in residents from nursing or personal care facilities. A county breakdown can be found here. All non-life-sustaining businesses are ordered to be closed and schools are closed statewide through the remainder of the academic year. Currently the entire state is under a stay-at-home order. Statewide - The Wolf Administration has since noon, April 26: Provided an update from Pennsylvania State Police on business closure enforcement actions. For the latest information for individuals, families, businesses and schools, visit "Responding to COVID-19" on pa.gov…* |
| **Schools and Education** | *Gov. Lee Unveils Safe Reopening Plan for Tennessee Schools Nashville, Tenn. - Tennessee Governor Bill Lee announced today the State of Tennessee's recommendations to reopen schools for the 2020-2021 school year. "Providing parents a choice in their children's education is incredibly important," said Gov. Lee. "In-person learning is the medically sound, preferred option. Our state is doing everything we can to work with local school districts and ensure that in-person learning is made available in a way that protects the health and safety of our students and educators, and this plan helps us accomplish that goal." "Leading health organizations, including the Centers for Disease Control and Prevention, American Academy of Pediatrics, and National Academies of Sciences, Mathematics, and Engineering, have all stressed the importance of in-person learning for students," said Tennessee Commissioner of Health Dr. Lisa Piercey. "The Department of Health has worked with Department of Education to establish a protocol to keep school buildings open safely and cause minimal disruption when positive cases occur." "Tennessee is prioritizing health and safety of our school communities," said Tennessee Commissioner of Education Penny Schwinn. "Ensuring schools, teachers, families, and students have the critical resources and supports they need to start the new school year strong is paramount, and I am thankful to Governor Lee for continuing to support education in Tennessee as schools reopen across the state." The recommendations from the Department of Health and the Department of Education are below: Health When to Test & Quarantine 10-Day Sick Window Anyone testing positive for COVID-19 must isolate themselves at home for 10 days from the onset of their symptoms or 10 days from the date their test was done if they never developed symptoms. Fever must be gone and they must be feeling better for at least 24 hours. 14-Day Quarantine Anyone who has been within 6 feet of someone who has COVID-19 for 10 minutes or more must quarantine themselves at home for 14 days from the last time they were with that person. These time periods do not change with a doctor's note or with a negative test. Contact Tracing Keeping schools open for in-person instruction depends upon our ability to quickly isolate people who are sick and quarantine their close contacts. If a child is ill, parents should not send them to school where they could infect others. If a child is diagnosed with COVID-19, parents are asked to assist the Department of Health by contacting the child's close contacts so those individuals can quarantine at home. If a parent is notified that their child has been in close contact of someone with COVID-19, please follow the guidelines and quarantine them at home for 14 days. Texting Platform Schools may be able to assist with notifying families of the need to quarantine through text messaging services. If parents receive a message from their child's school informing you that your child needs to stay at home for 14 days, please follow those instructions. Immunizations School entry immunizations have not changed.… 75 percent of students nationally receive mental health care in a school setting; In 2019, approx. 45,000 school-aged children were served for mental health through the community-based system; Approximately half of districts were able to address or check on wellness and safety of students during spring closures; Nearly 14 million students across the country go hungry when school is not in session, so resumption of in-person learning is critical to ensure access to nutrition. Academics Empowering Parents Whether it be in-person or virtual, we want parents to have a choice in their child's education. For those who choose the virtual option, the State will provide free resources to supplement their district's school-based services. The resources include: Early Literacy Resource: A free resource for students pre-K through 2nd grade to build foundational skills and support early literacy; PBS Learning Series: Complete lesson* |
| **Public Health Safety Behaviors** | *We have the first reported coronavirus case in Oregon, in Washington County. As a public health nurse, I want to make clear that we should not panic. Instead officials continue to recommend people in Oregon take everyday precautions to prevent the spread of many respiratory illnesses, including COVID-19 and influenza: Cover your coughs and sneezes with a tissue and then throw the tissue in the trash. Wash your hands often with soap and water for 20 seconds. If soap and water are not readily available, use an alcohol-based hand sanitizer that contains at least 60% alcohol. Avoid close contact with people who are sick. Avoid touching your eyes, nose and mouth with unwashed hands. Clean and disinfect surfaces that are often touched. Take care of your health overall. Staying current on your vaccinations, including flu vaccine, eating well and exercising all help your body stay resilient. Consult CDC's travel website for any travel advisories and steps to protect yourself if you plan to travel outside of the US.* |
| **Frontline / Essential Service Support and Volunteers** | *Get Tested Essential employees who directly interact with the public while working can get tested at one of the state's drive-through sites by calling the COVID-19 Hotline at 1-888-364-3065 or online at covid19screening.health.ny.gov Testing is free to individuals who are employed as health care workers, first responders, or in any position within a nursing home, long-term care facility, or other congregate care setting, including but not limited to: * Direct Care Providers * Firefighters * Medical Specialists * Nutritionists and Dietitians * Occupational/Physical/Recreational/Speech Therapists * Paramedics/Emergency Medical Technicians (EMTs) * Psychologists/Psychiatrists * Residential Care Program Managers * Laundry and Dry Cleaning Workers * Mail and Shipping Workers * Maintenance and Janitorial/Cleaning Workers * Retail Workers at Essential Businesses (e.g. * Grocery Stores, Pharmacies, Convenience * Stores, Gas Stations, Hardware Stores) * Security Guards and Personnel * Social Workers * Teachers/Professors/Educators * Transit Workers (e.g. Airports, Railways, Buses, and For-Hire Vehicles) * Trash and Recycling Workers Other essential employees include, but are not limited to: * Animal Care Workers (e.g. Veterinarians) * Automotive Service and Repair Workers * Bank Tellers and Workers * Child Care Workers * Client-Facing Case Managers and Coordinators * Counselors (e.g. Mental Health, Addiction, * Youth, Vocational, Crisis, etc.) * Delivery Workers * Dentists and Dental Hygienists * Essential Construction Workers at Occupied * Residences or Buildings * Faith-Based Leaders (e.g. Chaplains, Clergy Members) * Field Investigators/Regulators for Health and Safety * Food Service Workers * Funeral Home Workers * Hotel/Motel Workers * Human Services Providers Be sure, be safe, and thank you for your commitment and dedicated efforts to keep our nation running.* |
| **Family / Support Systems** | *This morning, I attended the funeral of an old friend that passed away after a tough battle with COVID. I say "old" friend, because I've known him a long time. We played baseball together, and we went hunting and fishing every chance we got when we were boys. But he wasn't old. He was 39. This afternoon, I visited our hospital and took some time to hear from the administrators and the ICU staff and nurses that are on the frontlines battling this virus. I saw how crowded that unit has become, and I heard about how tirelessly the doctors and nurses are working - and I could see the exhaustion and toughness in their faces. I listened to stories about patients that express their regret of not getting the COVID vaccine, and how emotional they become talking to their spouses, kids, and parents before being put on a ventilator - which is too often the last time their family hears their voice. Even those conversations are short and exhausting for the patient, because they can't keep oxygen levels up longer than saying a few "I love you's." It is a sad situation all around. This morning I hugged a mom and a widow at a funeral home and shook hands of brothers and friends as we talked about precious memories. Tomorrow, there will be more moms, dads, wives, husbands, brothers and sisters - and kids - that will be standing in funeral homes, greeting fellow grievers and sharing memories much sooner than they ever imagined. And there will be doctors and nurses that fought hard to care for those that died, grieving over every patient lost, but still working as the UV lights and room scrubbers come in to quickly make an empty room ready for the next person and the next family. Please join me tonight in praying for the families of those that have died and for those that are sick now. Please join me in praying for the doctors, nurses and staff that are caring for our family members and for our friends at all hours. This was a long and hard day, a day when I saw the effects of this terrible virus up close. The virus doesn't care about politics or personal beliefs. It just does its worst. Please do all you can to protect your family and friends…* |
| **Legislation** | *Weekly House Update- April 9, 2021 By the end of the 13th week of the 2021 Regular Session, more than 600 bills were signed into law. This week, the House voted in favor of a bill temporarily changing the deadline for filing and paying state income tax. SB593 extends the deadline this year to May 17, aligning the date with the recent extension issued for filing federal income tax. The also House voted in favor of the following bills addressing law enforcement, mental health, alcohol sales, and education: Law Enforcement HB1865-This bill requires all law enforcement officers in the state to complete annual training related to a law enforcement officer's duty to intervene if the law enforcement officer observes the use of excessive force by another law enforcement officer. HB1680-This bill states law enforcement agencies must assist a law enforcement officer involved in a critical incident in obtaining services that may help the officer recover from psychological effects. Mental Health HB1689-This bill will create an Arkansas Legislative Study on Mental and Behavioral Health. SB27-This bill requires the Arkansas Department of Health to ensure that the Suicide Prevention Hotline employs individuals who have experience working with veterans or are veterans. Alcohol Sales/Service Industry HB1748-This bill states that a referendum election to allow the sale of alcoholic beverages on Sunday may be called by a city or county if the governing body adopts a resolution by a two-thirds majority vote. SB479-This bill states that a restaurant with a valid alcoholic beverage permit from the Alcoholic Beverage Control Division (ABC) may expand its outdoor dining availability with approval from the municipality or county's local government zoning authority, which it is located without obtaining prior approval from the ABC. This bill also allows restaurants to remit sales taxes in quarterly payments rather than monthly for the next year. Education: SB160-This bill states that in the 2022-2023 school year, Holocaust education shall be taught in all public schools in a manner that generates an understanding of the causes, course, and effects of the Holocaust. SB524-This bill states that by August 1, 2022, each public school district and open-enrollment public charter school in the state shall prepare a three-year teacher and administrator recruitment and retention plan. The plan should include goals for recruitment and retention of teachers and administrators of minority races and ethnicities who increase diversity among the district staff and, at a minimum, reflect the racial and ethnic diversity of the district's students. SB394-This bill states that a public school district or open-enrollment charter school shall conduct a comprehensive school safety audit every three years to assess the safety, security, accessibility, and emergency preparedness of district buildings and grounds in collaboration with local law enforcement, fire, and emergency management officials…* |
| **Call for local action** | *RETURNING OUR ECONOMY & LIVES TO NORMAL RESPONSIBLY "The Opening of Alaska Following the COVID-19 Lockdown" By Senator Mike Shower, District E There is no question significant issues remain on our minds as we emerge from the COVID quarantine. Alaskans are eager to get back to work and reopen our economy, but we still grapple with finding a healthy equilibrium which addresses safety concerns, protects our constitutional rights, and rebuilds our economy. Some people have brushed off those who hold the notion our constitutional rights are of paramount importance during this pandemic. America's constitution was constructed in such a way to protect citizens from government. An argument can be made, as it recently was in Wisconsin's supreme court ruling, for the precise situation we find ourselves in today with governments mandating away certain liberties. Not all decisions made have to be government-driven priorities. You can make choices to protect yourself without being under the threat of government overreach. In many cases, the government solutions should be the last resort, not a first resource. I say this often; individual freedom is an inalienable, God-given right you have along with the responsibility attached to it. It isn't an arbitrary mandate that the government gets to authorize, or take away. Ultimately the 'best' scenario is for the current two-week quarantine to lift and for business to get back to the way things 'were.' We have gone so far down the current path it will take significant time to transition back to 'normal.' One thing is clear. We need to push decisions down to the most local level possible and stop assuming, from our limited viewpoints in government, that the State of Alaska can continue to force a one size fits all approach to getting Alaska back on track. We have entire regions of Alaska who've had zero cases of COVID and choosing to subject those areas to the same criteria as we see forced on places like Anchorage or even New York City is illogical and at the expense of the livelihoods of individuals. The State should provide guidance and support to our local communities. Still, Alaskans must not assume state government is best suited to address your individual communities' unique situation and challenges. I propose we open the discussion to citizens, especially at the local level, and decide there how to best get our economic engine up and running during our most productive time of year. If we miss this short seasonal window, our upcoming winter could be one of the 'darkest' periods on record since statehood. Can we afford to wait and see what comes next? I believe we must choose to be in the driver's seat and set a course for our future. Keeping Alaska locked up is an economic death sentence, the most ardent 'lock down' proponents can acknowledge this. There are many ways to proceed which solve this situation safely, unfortunately our government(s) have chosen many options which have inflicted the greatest economic harm. Why? Is embarking on a one size fits all approach which lacks due process and input from citizens most affected, the most appropriate way forward? As the State chose to shutter much of our economy, who bears the cost incurred by its actions and the negative impacts on Alaskans and Alaskan businesses? A few unemployment breadcrumbs or low-interest business loans do not come close to covering lost business. The state has fully funded itself (and then some), and its time for government to take a back seat at the trough and prioritize economic stimulus for the private sector. Two orders of business. Economic stimulus and government mandate relief. First. Much has been made of Norway's 'permanent' fund. They've seen fit to release over 4% of their fund to help citizens get through the COVID crisis. It's past time for the Alaskan legislature to open up the Earnings Reserve checkbook. We aren't borrowing money or racking up debt, but drawing from our savings account, to help offset some of the costs of what the government mandates caused…* |
| **Governmental Support** | *With State Coffers Flush, Tax Cuts and Infrastructure Dominate Senate Budget Proposal The Senate Budget sets in motion $12 billion in cash for infrastructure, capital projects over 10 years The Senate Budget includes sweeping tax package that reduces median household income tax payments by 37% in 2022 In addition to tax cuts and infrastructure, the proposal sets a $13 minimum wage for non-certified school employees and community college employees Provides salary increases and bonuses for all state employees Raleigh, N.C. - North Carolina's fiscal position is strong with state coffers flush because of a decade of prudent budgeting, and the Senate proposes to use the state's solid position to return a portion of surplus revenues to taxpayers and advance needed capital infrastructure projects. The Senate's proposed 2021-23 budget sets in motion a 10-year, $12 billion cash infrastructure and capital plan, including $3 billion in cash over the next two years for projects. The Senate's budget also includes a sweeping tax cut that reduces the personal income tax rate to 3.99% over five years and increases the zero-tax bracket to $25,500 for married filers. Senate Leader Phil Berger (R-Rockingham) said, "A decade of responsible budgets and growth-oriented tax policy has North Carolina in the best fiscal shape in a generation. This surplus came largely out of the pockets of North Carolina citizens and they deserve to see some of it returned to them." Details of the proposal are below. * The total proposed General Fund allocation is $25.7 billion in 2021-22 and $26.6 billion in 2022-23. * The total allocation of State Fiscal Recovery funds is $5.1 billion across the biennium. * The total State Capital Infrastructure Fund allocation is $4.3 billion over two years. * It replenishes the state's reserves, including the Rainy Day Fund. Capital/Infrastructure * Dedicates $4.3 billion to the State Capital and Infrastructure Fund (SCIF) over the next two years, $3 billion of which is available for projects ($1.3 billion is obligated to pay for previous debt). * Requires annual cash contributions to capital and infrastructure projects totaling $16.6 billion over 10 years, of which $12 billion can be used for infrastructure projects. (The remaining $4.6 billion will be put toward paying previous debts.) * Allocates $2.8 billion over the biennium for Strategic Transportation Investments. * Increases the General Maintenance Reserve by $250 million over the biennium to respond to storms and conduct routine maintenance. The reserve will be funded at a total of $1.4 billion over the biennium. * Invests more than $1.2 billion over the biennium for road resurfacing projects. * Provides more than $560 million over the biennium for the Bridge Program and over $140 million for bridge preservation. * Fully funds the UNC System's repairs and renovations request over the next four years, with $500 million allocated this biennium. * Provides $400 million to state agencies for repairs and major renovations. * Funds stream debris removal at $138 million over the biennium. * Funds construction of the Brody School of Medicine with $76 million. The total authorized cost of the project is $215 million. * Provides $55 million for NC State University's new STEM building. The total authorized cost is $80 million. * Funds the UNC-Pembroke Health Sciences Center with $32 million. The total authorized cost of the project is $91 million. * Provides $64 million over the biennium to Elizabeth City State University for a residence hall, sky bridge, dining facility, and flight school.…* |
| **Health Care Worker Testing and Mental Health** | *Update from the Gov's briefing yesterday afternoon: New Case Info as of yesterday at 5:45pm, April 24, 2020: Total positive cases in Arkansas: 2,741 (+276 since Thurs) o Includes 198 positives from Cummins Total active cases in Arkansas as of 4/24/2020: 1,763 Deaths: 46 (+1 since Thurs) Nursing homes: o Residents: 176 (+2 since Thurs) o Staff: 102 (+3 since Thurs) o Active investigations: 34 nursing homes Currently hospitalized: 104 (+3 since Thurs) Currently on ventilators: 25 (+1 since Thurs) Healthcare workers: 285 (+10 since Thurs) Recovered patients: 932 (+30 since Thurs) o Over one-third of total positive cases have recovered Total negative test results: 32,837 Total tests administered: 34,745 …Corrections facilities: o Forrest City Federal Corrections Institute: SS 79 inmates positive (+4) SS 11 staff positive (+1) SS Additional testing being done by CDC. o Arkansas Department of Corrections Cummins Unit: SS 690 inmates positive (+3 since yesterday) SS 35 staff positive SS ADH has nearly completed testing all inmates and are continuing to test additional staff Testing: o Continuing to encourage the testing surge. Please see attached link for map of testing sites: https://www.healthy.arkansas.gov/programs-services/topics/covid-19-guidance-for-getting-tested o Over 2,800 tests received Thurs- 4.1% positivity rate Lifting of some restrictions: o Future decision points for lifting restrictions: SS May 4- Target date for beginning to lift restrictions. SS April 29- Announcement on restaurants (in-service dining) SS April 30- Announcement on gyms/exercise facilities SS May 1-… Mental Health: Please see numbers below for mental health, addiction services and suicide prevention support: o Mental Health and Addiction Services: 1-844-763-0198 (Mon-Fri 8am-4:30pm) o National Suicide Prevention Lifeline: 1-800-273-8255 (24/7) Unemployment Insurance: o *Will be additional information 4/25/20 regarding…* |
| **Business / Economy** | *Here you go, Oklahoma!! We are opening up!! Open Up and Recover Safely (OURS) Oklahoma will begin implementing a three-phased approach to open Oklahoma's economy back up starting April 24, 2020. This statewide plan is: 1. Based on scientific modeling from public health experts 2. Intended to mitigate risk of resurgence 3. Intended to protect Oklahoma's most vulnerable citizens from the threat of COVID-19 ii 4. Intended for businesses and individuals to utilize in conjunction with guidance from the Oklahoma Department of Commerce, the Oklahoma State Health Department, and the Centers for Disease Control (CDC). Before proceeding to a new phase, the Secretary of Health and Mental Health will confirm: * That Oklahoma COVID-19 hospitalizations and incidents are at a manageable level, * that hospitals are treating all patients without alternate care sites, * that there is sufficient testing material in the state and ability to conduct contact tracing, and * that the state can quickly and independently supply sufficient Personal Protective Equipment (PPE) and critical medical equipment, including ICU equipment, to handle a surge Further, the State of Oklahoma must address the following core responsibilities before proceeding to Phase 1: * Ensure the ability to quickly set up safe and efficient testing for symptomatic individuals * Provide guidance intended to protect the health and safety of workers in critical industries o Employers should consider: Developing policies for temperature checks, sanitation, use and disinfection of common areas, and business travel Monitoring workforce for indicative symptoms; not allowing symptomatic people to physically return to work and consider implementing flexible sick leave and supportive policies and practices. Developing and implementing policies and procedures for workforce contact tracing following employee COVID-19 testing and implementing appropriate policies regarding social distancing and PPE, * Advise citizens regarding protocols for social distancing and face coverings o Individuals should: Continue to adhere to State and local guidance as well as complementary CDC guidance regarding social distancing Wash hands with soap and water Avoid touching the face Disinfect frequently-used items and surfaces as much as possible Consider using face coverings while in public and when using mass transit Stay home and contact their doctor if they are feeling sick * Monitor conditions to limit and mitigate any rebounds or outbreaks If hospital & incident rates remain at a manageable level for 14 days, we move to Phase 1 Phase 1 Individual guidance: * Continue following safer-at-home iv guidelines if they are over 65 or part of a vulnerable population * Maximize social distance from others when in public (e.g., parks, outdoor recreation areas, shopping areas) * Avoid socializing in groups or facilities that do not readily allow for appropriate social distancing * Minimize non-essential travel v and adhere to CDC guidelines and Executive Orders regarding isolation following travel Employer guidance: * Create plans to allow employees to return to work in phases * Close common areas or enforce social distancing protocols * Minimize non-essential travel and adhere to CDC guidelines and Executive Orders regarding isolation following travel * Honor requests of personnel who are members of a vulnerable population for special accommodations Specific Employer guidance: * Schools and organized sporting events and camps that are currently closed should remain closed until further notice * Visits to Senior Living Facilities and Hospitals are prohibited April 24, 2020, the following businesses can reopen: * Personal care businesses, such as hair salons, barbershops, spas, nail salons, and pet groomers can reopen for appointments only, must adhere to sanitation protocols, and follow guidelines posted on the Oklahoma Department of Commerce website regarding social distancing between customers and visitors at personal care businesses. * State parks and outdoor recreation should be reopened * Grocery stores should continue to maintain hours for vulnerable populations 2 May 1, 2020, the following businesses can reopen: * Dining, entertainment, movie theatres and sporting venues can operate using CDC- recommended social distancing and sanitation protocols. * Gyms can reopen if they adhere to CDC-recommended social distancing and sanitation protocols. * Places of worship can reopen for in-person meetings or worship if they leave every other row or pew open and adhere to CDC-recommended social distancing and sanitation protocols, plus the recommended guidelines from the Oklahoma Department of Commerce. * Bars should remain closed. * Tattoo Parlors can reopen for appointments only and must adhere to sanitation protocols and social distancing protocols for distancing between customers and visitors. If hospital & incident rates remain at a manageable level for 14 days…* |
| **Pandemic Time Course** | *Another tough day for the people of Massachusetts. The death toll rose to 154 (32 new deaths) in the Commonwealth. A woman in her 30's with pre existing conditions, a man in his 50's with unknown pre-existing conditions were the two under the age of 60 that passed. 2 men and 2 women in their 60's; 6 men and 2 women in their 70's; 8 men and 5 women in their 80's; 4 men and 1 woman in their 90's; most have which had pre-existing conditions, were the other people who succumbed to the virus today. . 4,870 people were tested and 1,228 came back positive. So far 56,608 people have been tested and 8,966 have come back testing positive for the coronavirus. Of those 8,966 positives 813 were hospitalized. Not a ton of new news today, but one piece was very sobering. Governor Baker discussed the latest modeling and predicted that the number of confirmed cases in Massachusetts will range between 47,000 and 172,000 during this epidemic. If the current fatality rate were to hold, that would mean between 705 and 2,580 COVID-19 deaths here in the Bay State. But remember from my call with Dr. Fauci yesterday - these are the projections given our current assumptions - it is in our hands to change these assumptions by being extra vigilant, a little more hand washing, a few less trips out, every little bit multiplied across the state. Just think, if every resident just took one less trip out a day (grocery store, coffee, whatever) that would mean nearly 7,000,000 less potential exposures. Extrapolate that out over the next month and we are looking at 200 million less opportunities for someone to spread the virus. So remember - we are all in this together and if we all think of the bigger picture - we can change the direction. Governor Baker ordered state beach parking lots to be closed since last weekend people were ignoring the social distancing guidelines and the no large group gathering order. (Really? this isn't that hard - and its not like it is the middle of Summer, ugh!) In addition, coastal parkways that provide access to state beaches will also be closed to both parking and dropping off passengers. Beaches will remain open for jogging, walking, fishing, etc - so let's hope we don't screw that up. On the positive side, some state parks will be opening earlier although parking will be limited to minimize congestion. Also, I'm sure you all heard - Bob Kraft flew the Patriots' team plane to China (no judgement, ok - a little judgement but I get it) and flew back with over a million N95 masks for our hospitals and first responders. This is huge especially after our shipment of 3 million masks (that we were able to get through BJ's - great Massachusetts company) were stolen - I guess officially seized but I call BS and I'm sticking with stolen, no matter if it was the feds or the mob - from the Port of New York. I hope there will be hell to pay on this one. In addition - over 500 new staffers were added to the state's unemployment agency to handle the call volume. This is still not enough as I and many other people have realized in trying to reach someone over there to change an application, check on status, etc. It is so frustrating and I can tell you that both myself and many of my colleagues are pushing for this to be fixed asap. I get that this hit them out of the blue - but we need better customer service - even if it is just internet-based updates. For those of you who are stopping by my page just to read the news for the day you can probably stop here. For the rest of you I hope you will indulge me a moment or two to share with you my thoughts on an issue that has been bothering me. Over the past few weeks, I have repeatedly expressed my frustration with the lack of transparency surrounding the reporting by the Massachusetts Department of Public Health and in particular their refusal to report successful outcomes of patients treated in area hospitals. I find this irresponsible on a variety of different levels. While I do not believe their claim that this information is not being reported to them, I do not feel they are nefarious in their intent. I know these people are working their asses off and are doing everything possible to protect us, and I suspect their rationale is that if they start reporting people being treated and released from hospitals, it will give the idiots amongst us the excuse they need to end their self-isolation and go about keeping this pandemic rolling. But besides my core belief that the government should always err on the side of transparency - there is a much more critical aspect that they are missing that needs to be addressed. Human beings are complicated, and we require more than basic needs to survive (dragging Maslow's hierarchy of needs out of the recesses of my brain, LOL) We must have love, friendship, companionship, a sense of being, goals, and most importantly hope. All the good stuff that makes life worth living…* |
| **Emergency Public Health Measures** | *I wanted you to see the governor's press release that followed his 1 pm Facebook Live press conference today. Many of his executive orders will bring relief while we work through this public health crisis. Gov. Holcomb Takes More Actions to Protect Hoosiers During Public Emergency Schools, Economy, Unemployment Insurance Changes Announced INDIANAPOLIS -- Governor Eric J. Holcomb took additional unprecedented actions today to protect and support Hoosiers during the COVID-19 outbreak by signing executive orders that extend the closure of schools, provide economic relief and protections for individuals and businesses, and expand unemployment insurance benefits for those impacted by job loss. "Every day we learn more about how to tackle this monster. We are being thoughtful about how to approach every action we are taking in this national public health emergency and putting Hoosiers' health and safety first," said Gov. Holcomb. Daily COVID-19 testing capacity in Indiana has expanded with the addition of a new partnership between the Indiana State Department of Health and Eli Lilly and Company, and at least one other entity has initiated testing this week. In the past 24 hours, about 200 tests have been completed. "As we increase the number of tests analyzed each day, no one should be caught off guard that the number of positive cases will increase," said Dr. Kris Box, state health commissioner. "This will help us know where community spread is occurring in Indiana and help us mobilize resources in affected areas." Here is a summary of covered actions. The Executive Orders, which contain additional actions, will be found at this link: https://www.in.gov/gov/2384.htm State of Emergency Extension The Governor will extend the current state of emergency an additional 30 days when it expires on April 5. K-12 schools All K-12 public schools will remain closed until May 1. Non-public schools are also ordered closed. This date may be revised to extend through the end of the 2019-2020 school year if circumstances warrant. All-state mandated assessments will be canceled for the current academic year. The governor has contacted U.S. Secretary of Education Betsy DeVos to share the state's plan and also has asked the Superintendent of Public Instruction Dr. Jennifer McCormick to pursue any federal waivers needed to cancel the requirements for accountability, chronic absenteeism and state-mandated assessments. Economy The state of Indiana will align with the federal government to delay state income tax payments from April 15 to July 15. The U.S. Treasury extended the deadline to pay federal income tax by 90 days. Penalties will be waived for 60 days for property tax paid after May 11. The state will work with counties that may experience cash flow stress because of the delay. The state will not immediately move forward with using $300 million in reserves to pay for several capital projects approved in the just-concluded legislative session and instead maintain flexibility to utilize the funds as needed for relief efforts and to maintain current services. The state will consider using bonding authority to move forward with the just-approved capital projects. Providers of essential utility services such as gas and electric, broadband, telecom, water and wastewater services are prohibited from discontinuing service to any customer during the public health emergency. The state's application to the U.S. Small Business Administration (SBA) was approved on Wednesday. This program provides targeted, low-interest loans of up to $2 million to help small businesses and nonprofits overcome the temporary loss of revenue as a result of coronavirus. See more at SBA.gov/Disaster. Unemployment Insurance Benefits The state will interpret Indiana's unemployment laws to the broadest extent possible to cover Hoosiers who are out of work because of COVID-19. Benefits will be paid to individuals who file their initial unemployment claims late. The Department of Workforce Development will allow individuals to continue to accrue unemployment eligibility if they take work leave because of COVID-19.… Housing No residential eviction proceedings or foreclosure actions may be initiated during the public health emergency. This does not relieve the individual of obligations to pay rent or mortgage payments. All public housing authorities are requested to extend deadlines for housing assistance recipients and required documentation to show eligibility for housing programs…* |
| **Debate Surrounding Public Policies** | *My bill (HB 1220) to allow pregnant minors to consent to their prenatal, delivery process, or postnatal care was heard in House Health and Human Services last night. It failed on a 6-7 vote. Currently in SD physicians may only give a minor any kind of prenatal care if delay would threaten a minor's life or health or if parents/guardians give consent. This law makes it difficult for a pregnant minor who wants prenatal care to receive it. Many pregnant teens arrive for prenatal care alone, often due to parents or guardians' inability to take time off from work or who are unsupportive of the pregnancy. This becomes a barrier to patients to receive necessary care. This bill would allow prenatal care to be given to pregnant minors and to choose an epidural to manage their pain if their parent or guardian aren't around to consent or if their parent/guardian withholds care. It's not uncommon for a minor to arrive at the hospital in labor & alone. They're scared, in pain, and want an epidural to manage pain, but can't receive it without a parent/guardian present to consent. For some, this means waiting hours until they arrives. Some parents/guardians never show. What's even more inconsistent with this situation is immediately following delivery, these same young women who may have been denied prenatal care or pain management are now entrusted and expected to consent to health care for their newborn. Healthcare professionals who testified spoke about the importance of prenatal care and how they are forced to deny care if a parent/guardian are absent or unwilling to consent. They said it's heartbreaking to deny care to pregnant girls and their unborn babies and it feels like they're breaking their Hippocratic Oath. They also spoke of the importance of epidurals and how helpless they feel when they can't help minors with their pain. Some physicians have seen brain damaged babies because women have pushed so long and have been denied an epidural. Opponents argued that our focus should be on parent/daughter communication and mending relationships. This is wishful thinking because sometimes relationships can't be mended, parents may be incarcerated, dealing with addictions/mental illnesses, or simply just not present in their daughter's life. Some parents will never be a part of their daughter's life and it's a terribly sad truth. Opponents also stated that parental consent is more important than giving minors consent. I argued that we're not trying to impact the family unit--we're trying to empower young pregnant mothers so they can have good healthcare outcomes for themselves and their babies. Every denial of care creates unnecessary risk to the patient, her pregnancy, and her baby. We must focus on supporting patients as they make informed decisions to enable a healthy pregnancy. I was disappointed we couldn't do that in committee last night…* |
| **Case Counts** | *DECEMBER 17, 2020 UPDATE "Yesterday, Extra Doses, Children & COVID Vaccinations, Canceled Unemployment Benefit, Teacher Vaccination Phase, Fed Rate Near 0, McGirt Offshoot Case" COVID-19 Oklahoma Test Results Positive Cases: 248,204 +2,975 Active Cases in OK: 31,770 -424 Currently Hospitalized: 1,699 (481 in ICU) Total Deaths: 2,144 +16 RECOVERIES: 214,290 +3,383 ICU Beds Available Statewide: 38 of 1,012 (4%) (Beds aren't the issue; the lack of staff to service those beds is the issue.) Antibody Tested: 101,297 Antibody Positive: 7,147 (7.1%) Region 3 Hospitalizations: 170 (-13) (Our region has the 3rd highest hospitalization rate in the state. Region 3 is an 18-county territory covering the southwest region of the state.) NEARBY COUNTIES (Key: # positive; # of deaths; # of recoveries \| # of active cases \| change) *Stephens County: 2350; 20; 1978 \| 352 active \| 6 change \| *22 hosp. @ DRH Stephens Co. Nursing Homes: 76;0;58 \| 18 active (epidemiology report data) Jefferson County: 337; 2; 278 \| 57 active \| 3 change \| 0 hosp. @ JCH Comanche County: 6030; 50; 5347 \| 633 active \| -263 change Cotton County: 359; 7; 305 \| 47 active \| -1 change Grady County: 3369; 33; 3046 \| 290 active \| -14 change Carter County: 2231; 16; 1905 \| 310 active \| 10 change Garvin County: 1928; 18; 1675 \| 235 active \| 19 change Love County: 761; 1; 670 \| 90 active \| 1 change LOCAL MUNICIPALITIES (Key: # positive; # of deaths; # of recoveries \| # of active cases Duncan: 1466; 12; 1247 \| 207 active \| 7 change Addington: 5; 0; 5 \| 0 active \| 0 change Comanche: 232; 4; 196 \| 32 active \| 2 change Hastings: 17; 0; 14 \| 3 active \| 0 change Loco: 28; 0; 25 \| 3 active \| -1 change Ringling: 93; 1; 76 \| … -124 change Pauls Valley: 667; 5; 572 \| 90 active \| 5 change Sterling: 40; 0; 35 \| 5 active \| 1 change Stillwater: 3920; 12; 3548 \| 360 active \| -3 change Sulphur: 588; 5; 491 \| 92 active \| -7 change Temple: 89; 5; 70 \| 14 active \| 2 change Thackerville: 82; 0; 73 \| 9 active \| -1 change Tulsa: 25068; 213; 22088 \| 2767 active \| -71 change Wilson: 151; 1; 139 \| 11 active \| -19 change DAILY BRIEFING -Oklahoma experienced 3,238 new cases and 42 more deaths related to the virus yesterday. The most deaths reported thus far are 54 deaths on Dec. 2. The state's seven-day rolling average of new cases is 3,176. Overall, cases are trending up, again, which concerns our hospitals. -OK received 40,000 doses of vaccine.…* |
| **Long-term Care Facilities** | *Today, I joined with the Connecticut Senate Republican Caucus in asking the governor to reconsider his administration's policies on nursing home visitation to allow for safe indoor family visits for ALL residents, not just residents who are near death or who have recently declined. We noted to the governor that the Connecticut Department of Public Health's recent order does not allow for visitation for residents who are not near death and who would prefer to have an in-person visit indoors due to reasons such as mobility issues. The order ONLY allows for indoor visits when a resident is near death or has seen a significant decline in health. This ignores the importance of family visits in preventing deterioration of physical and mental health. The DPH order also does not address what happens when the weather turns colder in a few months and outdoor visits can no longer occur. We want the governor to expand the DPH order so that family visits can be prioritized for all nursing home residents. Family members and loved ones are the 'eyes and ears' of elderly individuals, especially for those suffering from Alzheimer's disease or dementia. When a person with dementia does not have the ability to interact with their loved ones and what is familiar to them, their capacity diminishes, further accelerating decline. Forcing a person to wait until their capacity diminishes to see their family is the complete opposite strategy of patient centered care we should be embracing. With family members not being allowed to visit, other health issues can go unnoticed and worsen. I will keep you posted on this developing situation.* |
